# Molecular mechanisms of Charcot-Marie-Tooth neuropathy linked to mutations in human myelin protein P2

**DOI:** 10.1038/s41598-017-06781-0

**Published:** 2017-07-26

**Authors:** Salla Ruskamo, Tuomo Nieminen, Cecilie K. Kristiansen, Guro H. Vatne, Anne Baumann, Erik I. Hallin, Arne Raasakka, Päivi Joensuu, Ulrich Bergmann, Ilpo Vattulainen, Petri Kursula

**Affiliations:** 1 Faculty of Biochemistry and Molecular Medicine, University of Oulu, 90220 Oulu, Finland; 2 Department of Physics, Tampere University of Technology, 33720 Tampere, Finland; 3 Department of Biomedicine, University of Bergen, 5020 Bergen, Norway; 4 Division of Psychiatry, Haukeland University Hospital, 5021 Bergen, Norway; 5 Department of Sustainable Chemistry, Technical Faculty, University of Oulu, 90570 Oulu, Finland; 6 Biocenter Oulu, University of Oulu, 90220 Oulu, Finland; 7 Department of Physics, University of Helsinki, 00560 Helsinki, Finland

## Abstract

Charcot-Marie-Tooth (CMT) disease is one of the most common inherited neuropathies. Recently, three CMT1-associated point mutations (I43N, T51P, and I52T) were discovered in the abundant peripheral myelin protein P2. These mutations trigger abnormal myelin structure, leading to reduced nerve conduction velocity, muscle weakness, and distal limb atrophy. P2 is a myelin-specific protein expressed by Schwann cells that binds to fatty acids and membranes, contributing to peripheral myelin lipid homeostasis. We studied the molecular basis of the P2 patient mutations. None of the CMT1-associated mutations alter the overall folding of P2 in the crystal state. P2 disease variants show increased aggregation tendency and remarkably reduced stability, T51P being most severe. In addition, P2 disease mutations affect protein dynamics. Both fatty acid binding by P2 and the kinetics of its membrane interactions are affected by the mutations. Experiments and simulations suggest opening of the β barrel in T51P, possibly representing a general mechanism in fatty acid-binding proteins. Our findings demonstrate that altered biophysical properties and functional dynamics of P2 may cause myelin defects in CMT1 patients. At the molecular level, a few malformed hydrogen bonds lead to structural instability and misregulation of conformational changes related to ligand exchange and membrane binding.

## Introduction

A multilamellar membrane structure, myelin, enwraps selected axons in the nervous system, enabling the prompt transmission of nerve impulses along the axonal membrane. Myelin contains a compact lipid-rich compartment with a unique set of membrane-associated proteins, such as myelin protein zero (MPZ), peripheral myelin protein of 22 kDa (PMP22), myelin basic protein (MBP), and myelin protein P2, and more loosely packed regions that also encompass cytoplasm. Close interplay between myelin proteins is important for the formation of cytoplasmic channels within myelin^1^. In the peripheral nervous system (PNS), Schwann cells envelop axons and form insulating myelin sheaths. Defects in this fundamental structure result in chronic, severe neuropathological conditions, affecting nerve conduction velocity and neuron viability.

Charcot-Marie-Tooth disease (CMT) is one of the most common inherited human neuropathies (prevalence of 1:2500), affecting both motor and sensory nerve conduction in the PNS^2^. Several types of CMT exist; the most common form, autosomal dominant CMT1, influences Schwann cells and myelin. In CMT1, motor and sensory nerve conduction velocities are remarkably reduced, leading to muscle weakness and atrophy in the feet and occasionally also in upper limbs. Demyelination and onion bulb formation are present in most CMT1 patients. Most commonly, CMT1a is caused by a duplication of or a point mutation in the *pmp22* gene, and 70–80% of all CMT1 cases are associated with *pmp22* mutations^3^. CMT1b results from mutations in the *mpz* gene and comprises 4% of all CMT1 cases. A number of other CMT1-associated genes have been described^2^. Recently, three point mutations in the *pmp2* gene, coding for the myelin P2 protein, were linked to autosomal dominant CMT1. The I43N mutation was discovered in two families^4,5^ and T51P and I52T each in one family^6^. All patients with these mutations have reduced motor and sensory nerve conduction velocities, thinner myelin sheaths, and demyelinating axons with onion bulb formation. Symptoms also include progressive hand and foot muscle weakness and atrophy, as well as foot deformity and sensory loss in limbs^4–6^. Although the pathological features of different CMT1 types are comparable, the molecular pathomechanisms are different^7^.

P2 is mainly expressed by Schwann cells in the PNS^8^. It is located in compact myelin, stabilizing the multilayered lipid membrane assembly. P2 is a small 15-kDa protein with a β barrel structure covered by an α-helical lid^9,10^. It belongs to the conserved family of fatty acid-binding proteins (FABPs) and can transfer fatty acids from and to lipid membranes using a collision transfer mechanism, indicating a functional role in the lipid homeostasis of myelin^11^. P2 may also bind cholesterol; a CRAC cholesterol binding motif is found at its C terminus^9,12^.

Here, we show that the crystal structures of CMT1-associated P2 mutant forms remain nearly unaltered, but the stability of the mutant proteins is remarkably reduced. Mutant proteins show decreased solubility, T51P triggering the most significant effect. T51P has a reduced membrane stacking activity, while I43N and I52T maintain their ability to bind and stack lipid membranes. On the other hand, protein dynamics, as well as fatty acid and membrane binding kinetics, of all P2 mutants differ from those of wild-type P2 (P2wt). The mutations may cause defects in protein folding and the regulation of functional conformational changes, and they provide clues into ligand binding dynamics in the FABP family.

## Results

In order to explore the functional and structural effects of CMT1-linked mutations in P2, we expressed and purified all three mutant proteins; I43N, T51P, and I52T. We used a range of experimental techniques together with atomistic simulations to study the effects of the mutations on P2 structure, function, and dynamics.

### CMT mutations increase P2 aggregation but do not affect the overall crystal structure

**Figure 1:**
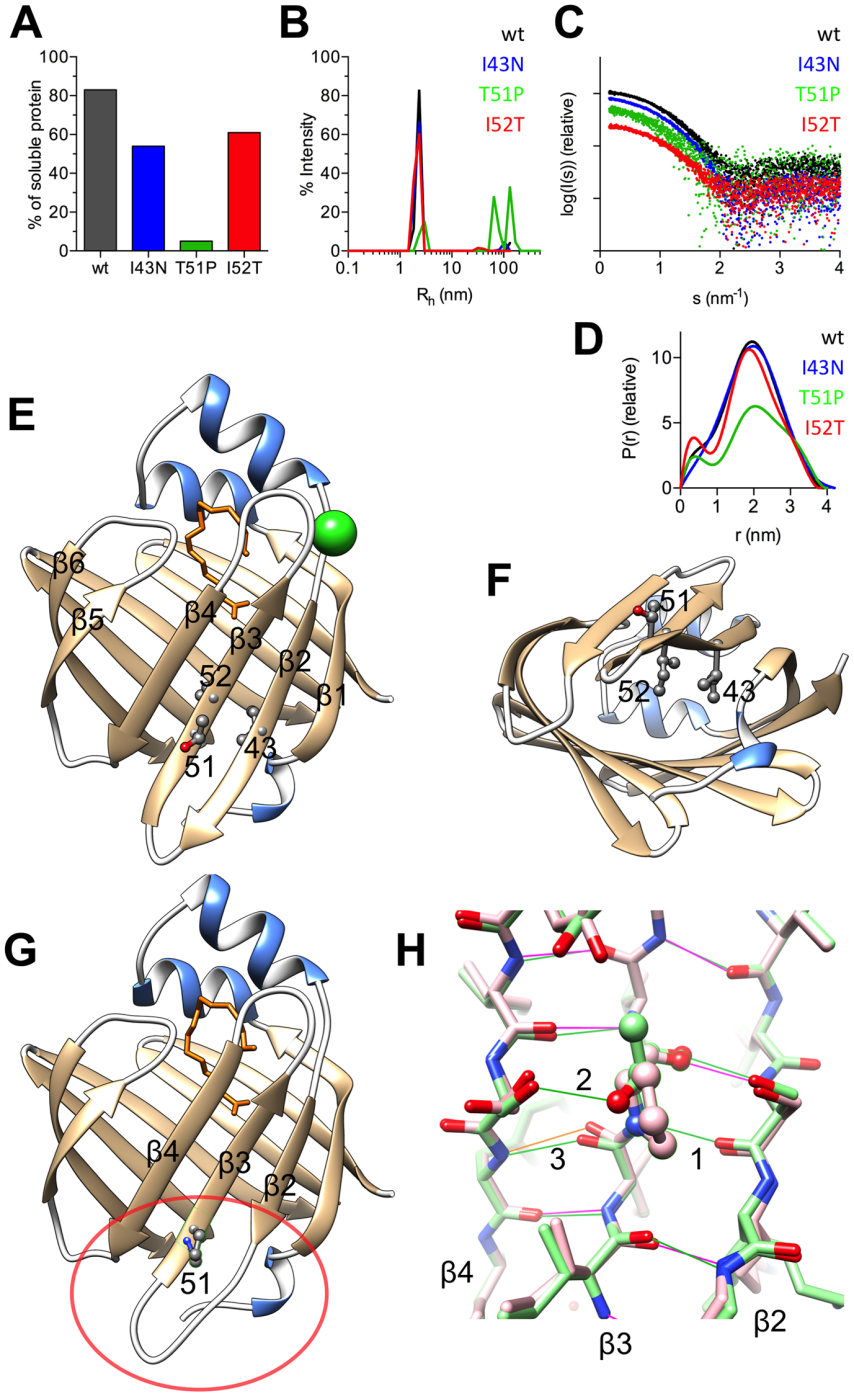
Structure and stability of P2. (**A**) Solubility analysis. (**B**) Dynamic light scattering indicates aggregation of T51P. Note that the curves for P2wt and I43N nearly overlap in panels B and D. (**C**) The SAXS scattering curve. (**D**) Distance distribution functions. (**E**) Crystal structure of P2 with the mutant positions labelled. The bound fatty acid is shown in orange and the anion binding site with a chloride ion (green). (**F**) The mutation hot spot viewed from the bottom. (**G**) The crystal structure of T51P. Note the decreased area of the β sheet close to the mutation; compare the circled area with the corresponding area in panel E. (**H**) Hydrogen bonds near residue 51 in wild-type (green) and T51P (pink); the shown region corresponds to the circled area in panel G. Three hydrogen bonds are labeled: 1) main-chain H bond that is lost in the mutant protein, 2) side-chain hydrogen bond lost in the mutant protein, 3) main-chain H bond that is in an unfavorable conformation in the mutant protein (orange).

In a prokaryotic expression system, all mutants showed a higher tendency to form insoluble aggregates than P2wt (Fig. 1A). Only ~5% of T51P was soluble, despite optimised cell lysis conditions. Also both I43N and I52T showed reduced solubility compared to P2wt. However, soluble forms of all mutants could be purified and eluted as a single monomeric peak in size exclusion chromatography (SEC). We used dynamic light scattering (DLS) to further investigate the aggregation tendency of P2. P2wt, I43N, and I52T remained 100% monomeric for 24 h after SEC, while T51P was 98.3% monomeric, and high-molecular-weight forms were already present (Fig. 1B). The hydrodynamic radius (R_h_) of monomeric T51P was also larger compared to the other variants (Table 1).

**Table 1:** DLS, SAXS, and MD simulation parameters.

Protein	DLS		SAXS			MD simulations	
	R_h_ (nm)	Monomeric (%)	R_g_ (nm)	D_max_ (nm)	V_porod_ (nm^3^)	R_g_ apo (nm)	R_g_ holo (nm)
wt	2.11	100	1.45	3.9	14	1.45	1.44
I43N	2.18	100	1.55	4.0	18	1.43	1.44
T51P	2.40	98.3	1.56	4.2	13	1.50	1.45
I52T	2.22	100	1.45	3.9	12	1.43	1.45

Small-angle X-ray scattering (SAXS) data were collected to monitor possible variation in the solution structures of the patient mutants (Fig. 1C). Scattering patterns of all mutants resemble those of P2wt. Nevertheless, the radius of gyration (R_g_) and maximum dimension (D_max_) of I43N and T51P slightly deviate from P2wt, while I52T gave similar results (Table 1). The shape of the distance distribution function (Fig. 1D) differs between the variants, T51P and I52T being most divergent. This could be related to the position of the α-helical lid, the overall structure of the β barrel, and/or the level of saturation with fatty acid inside the barrel.

To address structural consequences of the mutations, we determined the respective crystal structures at high resolution (Table 2). All mutated residues are well defined in electron density. The mutations are located in the middle of the strands β2 and β3, in close proximity to the core of the β barrel (Fig. 1E,F). However, the overall fold of the structures is nearly identical to P2wt, with RMS deviations of 0.1–0.2 Å (Cα atoms). Considering the differences in solution conformations, this is a reflection of the selection of a single low-energy conformer into the crystal lattice, closely resembling the wild-type crystal structure. All structures present a gap in the β barrel between the strands β4 and β5, with no hydrogen bonds formed between the strands. This is a common feature of the FABP family^13–17^, and it is noteworthy that all the CMT1-linked P2 mutations lie close in space to this gap (Fig. 1E). The largest difference is triggered by the T51P mutation, which decreases the area of the β sheet β1–4 (Fig. 1G). The mutation hinders the carbonyl group of Ile50 from forming a hydrogen bond with the amino group of Phe65 in the β4 strand; Pro51 also cannot form a hydrogen bond to Ser44 in the strand β2 (Fig. 1H). Two water molecules in close proximity to the residue 51 and the β2 and β3 strands are not found in the crystal structure of T51P, but they exist in all other structures. I43N and I52T do not alter the main chain conformation, and neither side chain generates steric clashes. These changes from a non-polar to a polar residue do not induce large structural changes in the crystal structure. However, the amino group of the Asn43 side chain of I43N forms an extra hydrogen bond to the main chain carbonyl group of Phe5, located at the end of the short α helix at the N terminus of P2.

**Table 2:** Crystallographic data collection and structure refinement.

Mutant	I43N	T51P	I52T
*Data collection*			
Beamline	I04/Diamond	I03/Diamond	I04/Diamond
X-ray wavelength (Å)	0.9795	0.9762	0.9795
Space group	P41212	P41212	P41212
Unit cell dimensions a, b, c (Å)	65.0,65.0,101.1	66.0,66.0,100.9	64.8,64.8,101.1
α, β, γ (°)	90,90,90	90,90,90	90,90,90
Resolution range (Å)	46–1.59 (1.69–1.59)	46–1.72 (1.87–1.72)	46–1.53 (1.63–1.53)
No. unique reflections	29518 (2710)	44944 (7154)	33053 (3233)
Completeness (%)	98.7 (89.2)	99.6 (97.5)	99.6 (99.6)
Redundancy	6.6 (6.4)	6.1 (6.1)	6.0 (5.9)
R_sym_ (%)	6.7 (300)	7.4 (267)	5.1 (265)
R_meas_ (%)	7.0 (327)	8.1 (292)	5.3 (257)
<I/σI>	18.4 (0.7)	12.8 (0.8)	22.0 (0.7)
CC_1/2_ (%)	100 (73.4)	99.9 (67.8)	100 (72.8)
Wilson *B* (Å^2^)	29.3	31.2	29.2
*Structure refinement*			
R_cryst_/R_free_ (%)	18.4/21.4	16.9/19.1	17.0/20.1
RMSD bond lengths (Å)	0.007	0.006	0.007
RMSD bond angles (°)	1.0	1.0	1.1
Molprobity score	1.24	1.57	1.97
Ramachandran favoured/ outliers (%)	100/0	98.7/0	100/0

### Mutated forms of P2 have reduced stability

**Figure 2:**
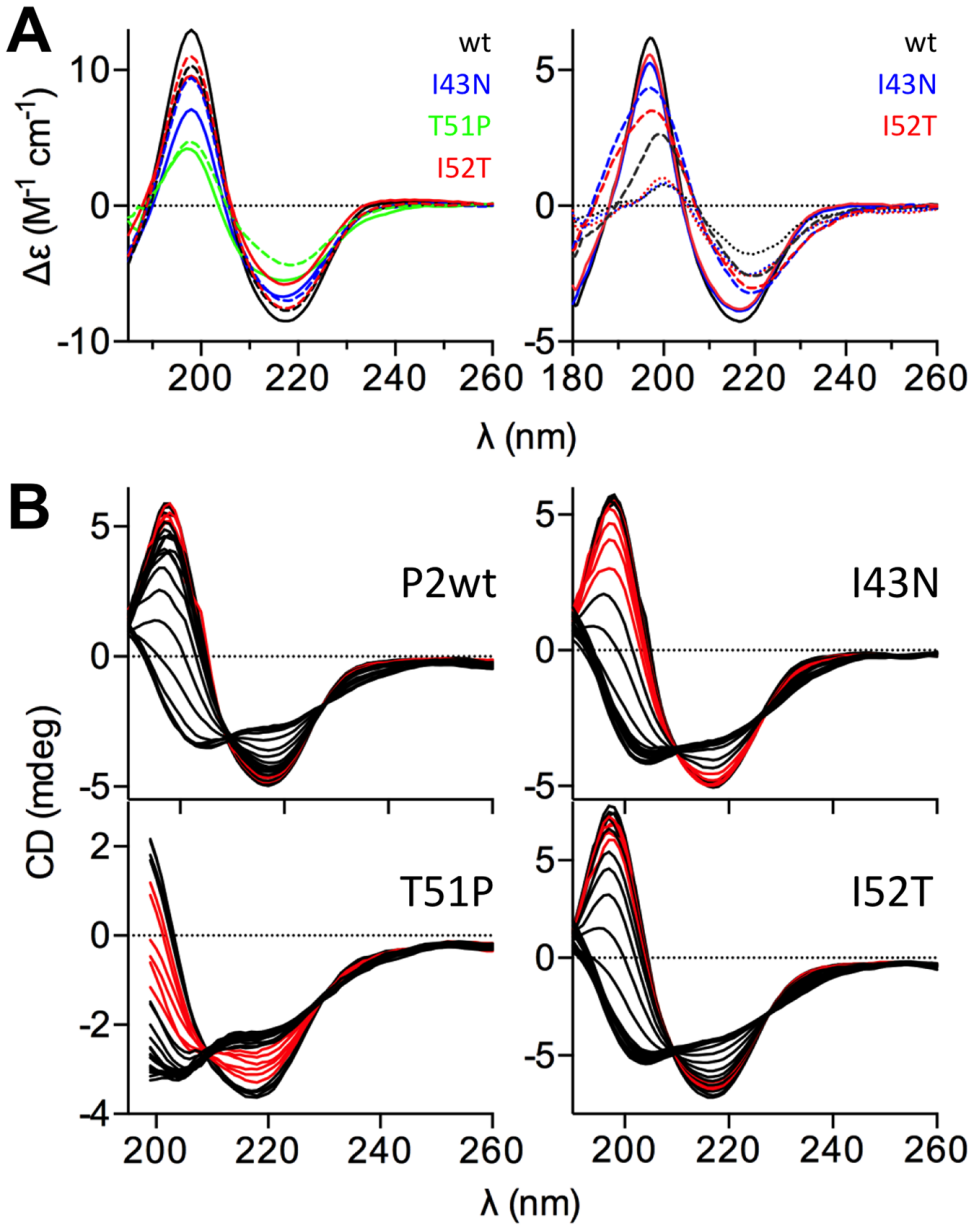
SRCD analysis of P2 folding and membrane interactions. (**A**) Left: SRCD in phosphate buffer with and without DMPC:DMPG vesicles. Right: SRCD in H_2_O and with DMPC:DMPG (dashed lines) and DOPC:DOPG (dotted lines) vesicles. (**B**) Temperature scans of all variants. Temperatures between +40–50 °C are shown in red for clarity.

Due to increased aggregation tendency, the folding of the P2 variants was examined using synchrotron radiation circular dichroism spectroscopy (SRCD). Spectra were recorded in water and in phosphate buffer (Fig. 2A). The intensity of both the positive (at 198 nm) and negative (at 218 nm) peaks varied, P2wt giving the highest and T51P the lowest signal, suggesting formation of soluble aggregates of the mutant proteins. Minor variations were also observed in the shape and x axis intersections of the spectra, mainly for T51P, indicative of lower secondary structure content or altered conformation.

In the presence of 1,2-dimyristoyl-*sn*-glycero-3-phosphocholine (DMPC): 1,2-dimyristoyl-*sn*-glycero-3-(phospho-*rac*-(1-glycerol)) (DMPG) or 1,2-dioleyl-*sn*-glycero-3-phosphocholine (DOPC): 1,2-dioleyl-*sn*-glycero-3-phosphatidyl-*rac*-glycerol (DOPG) vesicles, the spectra of P2 clearly changed (Fig. 2A). This phenomenon is more pronounced in water (Fig. 2A, right), where competition between lipid head groups and phosphate does not exist. In phosphate buffer (Fig. 2A, left), the positive peak at 198 nm decreased with P2wt and I52T, while I43N and T51P show opposite behavior. In water, the shape of the P2wt, I43N, and I52T spectra change considerably upon adding lipid vesicles, especially when DOPC:DOPG vesicles are introduced.

We further studied the thermal stability of P2 mutants using conventional CD spectroscopy (Fig. 2B). T_m_ analysis and CD spectra illustrate a drastic reduction of the stability of all patient mutants compared to P2wt (T_m_ = +65 °C); T51P has the lowest T_m_, +41 °C, and already at +38 °C, the conformation of T51P changed and unfolding started. I43N has a T_m_ at +48 °C and I52T at +52 °C. Therefore, both of these mutants also have decreased thermal stability compared to P2wt.

### Patient mutations modify the dynamics of all P2 mutants

**Figure 3:**
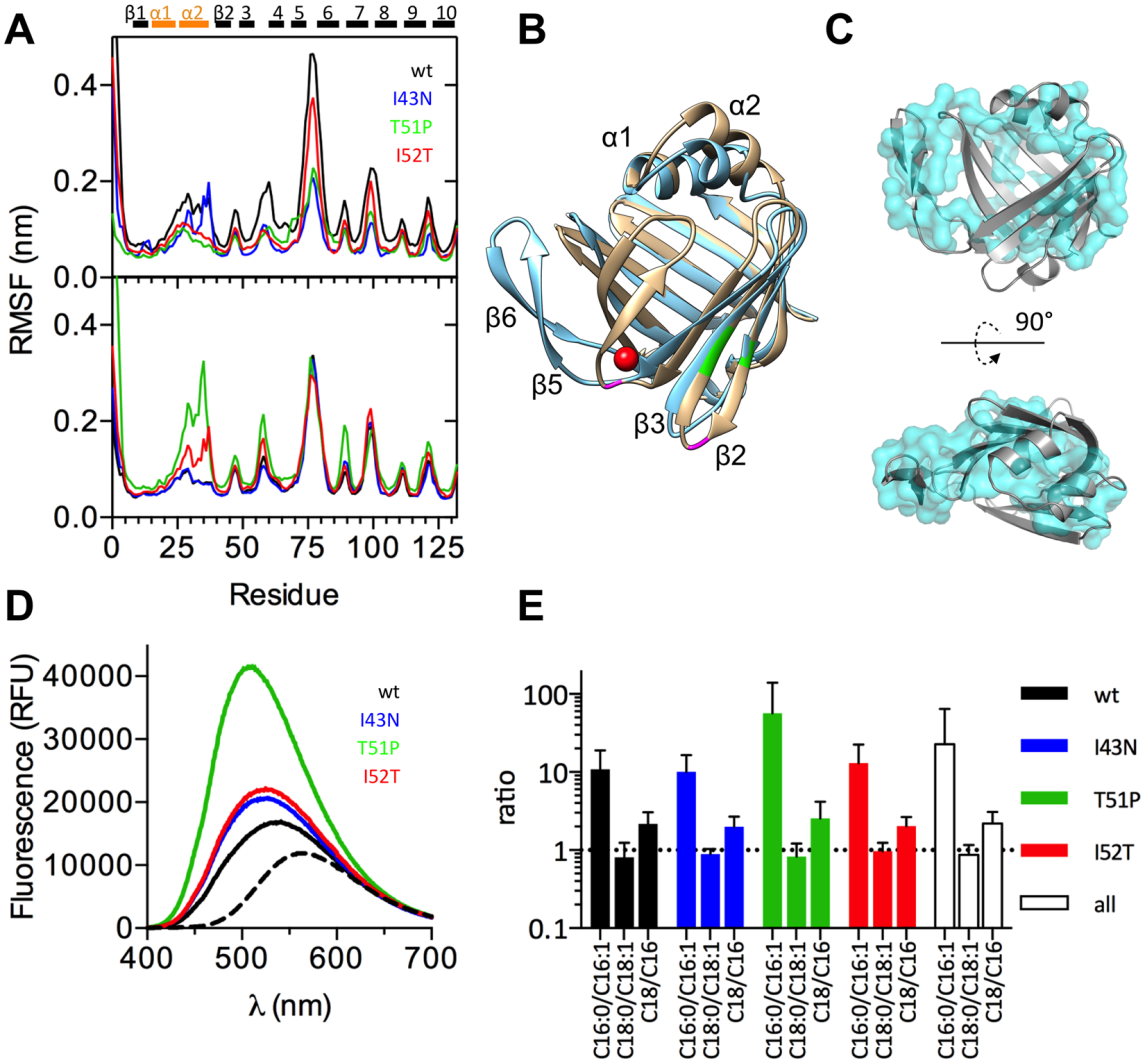
Dynamics, open conformation, and fatty acid binding. (**A**) RMS fluctuation of apo (top) and liganded (bottom) forms. Above the graphs, the secondary structure elements of P2 are shown (black, strand; orange, helix). (**B**) Structure of P2wt (light brown) superimposed on the open conformation of T51P seen in simulations (blue). The two conserved FABP Gly residues in the β2-β3 and β4-β5 loops are shown in magenta, the positions of the mutations in green, and the “structural” water molecule bound inside the β4-β5 loop in FABPs in red. (**C**) Comparison of the open conformation (cartoon) and the SAXS envelope from T51P data (blue transparent surface) from two orientations. (**D**) Fluorescence spectra of DAUDA with 10 µM proteins. (**E**) LC-MS fatty acid analysis. All P2 variants prefer either palmitate (C16:0) or 18-carbon fatty acids (C18:0 and C18:1) Recombinant P2 carries more C18 than C16 fatty acids.

Root mean square fluctuation (RMSF) analysis from atomistic molecular dynamics (MD) simulations was used to examine P2 dynamics with and without bound palmitate inside the β barrel. Palmitate-bound P2wt is generally more dynamic than its empty counterpart. The most mobile parts of P2wt are located in the loops and, to some extent, in the helical lid (Fig. 3A); these segments correspond to the portal region of FABPs. Similar behaviour was seen with palmitate-bound I43N. In MD simulations, palmitate-bound T51P is overall much more dynamic than the other P2 variants and shows strong fluctuations in the lid region as well as in the β sheets β2-5 and the loops connecting them. The lid region and the β3-β4 loop of I52T also fluctuate more heavily than those of P2wt. However, when the fluctuations of ligand-free P2 are compared, P2wt seems to be the least dynamic variant. I52T shows some fluctuations at certain parts, while I43N and T51P are rather rigid. Surprisingly, T51P is the least dynamic of the apo forms (Fig. 3A). The apo form of T51P is overall less dynamic than palmitate-bound T51P. The lid region of I52T and the β5-β6 loop of I43N are also destabilised by palmitate, while other parts of the mutants show reduced fluctuation upon palmitate binding.

In simulations of P2, especially in the case of apo T51P, opening of the β barrel at the gap is observed (Fig. 3B). The hinge for this movement lies at Gly68 in the β4-β5 loop, which is one of the two absolutely conserved Gly residues across all human FABPs^10^. At the hinge also lies a buried water molecule, which is conserved in FABPs and was suggested to be relevant for correct folding^18^. It is possible that the conserved Gly residues and the water molecule also play roles in functional conformational changes in FABPs, including P2. R_g_ for all eight systems (four P2 forms, +/−palmitate) was estimated based on the MD simulations. The obtained values (Table 1) are in good agreement with those derived from SAXS. *Ab initio* modeling of T51P based on the SAXS data provides a shape very similar to that observed in the MD simulation (Fig. 3C), confirming the presence of an open, more extended conformation, of P2 in solution. In addition, multicomponent analysis of the SAXS data indicates a 7% fraction of open conformation of T51P in solution, while the other P2 forms exist in the closed conformation.

We also calculated the number of water molecules inside the P2 barrel during each simulation. The average number of water molecules inside unliganded P2 was 27.7, 30.5, 23.7, and 30.4 (for P2wt, I43N, T51P and I52T, respectively). For palmitate-bound P2 forms, the respective numbers of water molecules were 21.0, 22.1, 29.1, and 22.0. In both cases, T51P differs from the other variants, interestingly having less water molecules within the β barrel without than with palmitate inside. Here, the behaviour of the Ile mutants resemble each other but slightly differ from P2wt. These values are in accordance with studies on FABP, whereby faster exchange of water was observed for liganded FABP than for apo-FABP^19^ - a major route for water flow between bulk solvent and the internal cavity was suggested to be the gap between the strands β4 and β5. Both the apparent lower water content of apo T51P as well as its low RMSF during the MD simulation can be explained by the observation that the unliganded T51P structure opens up early in the simulation and remains in the open conformation.

### Fatty acid binding in P2 CMT mutants

P2 belongs to the family of FABPs, and in all crystal structures of human P2, there is a fatty acid inside the β barrel^9,10,20,21^. Also in the crystal structures of all mutant proteins studied here, a fatty acid, modelled as palmitate, is visible inside the β barrel. P2 captures abundant fatty acids during expression in *E. coli*^9,10^. With our earlier atomic-resolution crystallographic data, we modelled the fatty acid component as a mixture of palmitate and *cis*-vaccenate^10^.

To study fatty acid binding, we performed end-point binding assays with a fluorescent fatty acid derivative probe, 11-dansylaminoundecanoid acid (DAUDA)^22^. The peak maxima clearly increase and shift in the presence of P2, indicating an interaction involving a non-polar environment between DAUDA and all P2 variants (Fig. 3D). This phenomenon is most pronounced with T51P, but also I43N and I52T show higher fluorescence than P2wt. In this experimental setup, T51P seems to bind to DAUDA more effectively than the other mutants or P2wt, which might be related to its higher tendency to open.

Since the structure and dimensions of DAUDA diverge from natural fatty acids, we used liquid chromatography-mass spectrometry (LC-MS) to investigate the fatty acids bound to P2. Fatty acids with 16–20 carbons without or with a double bond were monitored. The total amount of bound fatty acids was similar between all the P2 variants, and C16 and C18 fatty acids together comprise nearly 100% of the total fatty acid content. We compared the amount of saturated and non-saturated C16 and C18 fatty acids, and noted the fatty acid content of all variants was nearly identical (Fig. 3E). P2 contains more C18 (60–70%) than C16 fatty acids (30–40%). Palmitate is the preferred C16 ligand, while for C18, nearly equal amounts of saturated and non-saturated fatty acid are observed. The result validates our high-resolution model^10^, in which we earlier built in palmitate (C16:0) and *cis-*vaccenate (C18:1) with partial occupancies.

### P2 binding to immobilised lipid vesicles

**Figure 4:**
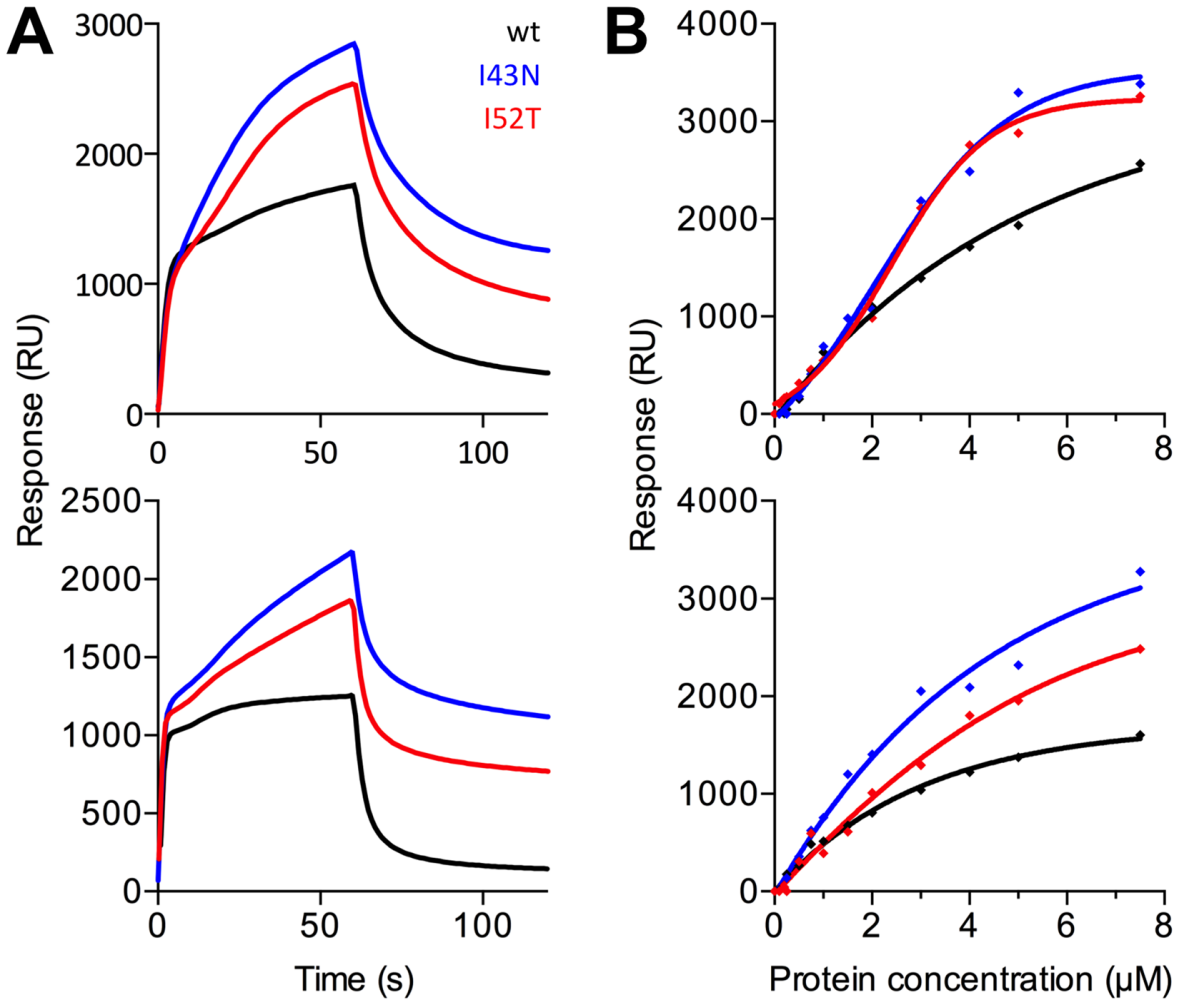
Membrane surface binding. (**A**) DMPC:DMPG (top) and DOPC:DOPG (bottom) sensorgrams with injection of 4 µM protein. (**B**) DMPC:DMPG (top) and DOPC:DOPG (bottom) fits for a protein titration.

The binding of P2 onto lipid membrane surfaces was studied using surface plasmon resonance (SPR) with two different lipid compositions, differing only in the identity of the hydrocarbon chains of the lipids (Fig. 4). SPR was not carried out with the T51P mutant due to its tendency to aggregate. P2wt and the I43N and I52T mutants all bound to membranes with affinities in the 2–6 µM range, irrespective of the lipid composition (Table 3). Despite similar apparent affinity, the kinetics of the wild-type and mutant proteins were different, and in fact, more of the mutant proteins eventually bound to the membrane, giving a continuous increase in response during the injection (Fig. 4A). This could be a sign of aggregation of the mutants onto the lipid bilayer after initial binding. P2wt, on the other hand, bound rapidly to the membrane, reaching a plateau by the end of the injection, and dissociated fast. All these features were similar between membranes made of DMPC:DMPG and DOPC:DOPG. The result is an indication of possible membrane-induced aggregation of mutant P2 forms. The behaviour of the P2 mutants resembles somewhat the kinetics shown by another major myelin protein, MBP, which folds onto the membrane surface, forming an amorphous phase of protein, and dissociates very slowly^23,24^.

**Table 3:** Membrane surface affinity of P2 deduced from SPR data. The values are apparent K_d_ in µM, based on two separate experiments.

P2 variant	P2wt		I43N		I52T	
Lipid	DMPC:DMPG	DOPC:DOPG	DMPC:DMPG	DOPC:DOPG	DMPC:DMPG	DOPC:DOPG
Two-state reaction	6.03 ± 1.05	2.44 ± 0.54	3.77 ± 0.11	3.48 ± 2.64	6.37 ± 1.01	2.53 ± 1.28
Sigmoidal model	4.78 ± 0.45	2.59 ± 0.62	3.25 ± 2.69	5.24 ± 1.22	0.95 ± 0.06	3.12 ± 0.65

### All mutants stack lipid membranes, but T51P is less active

**Figure 5:**
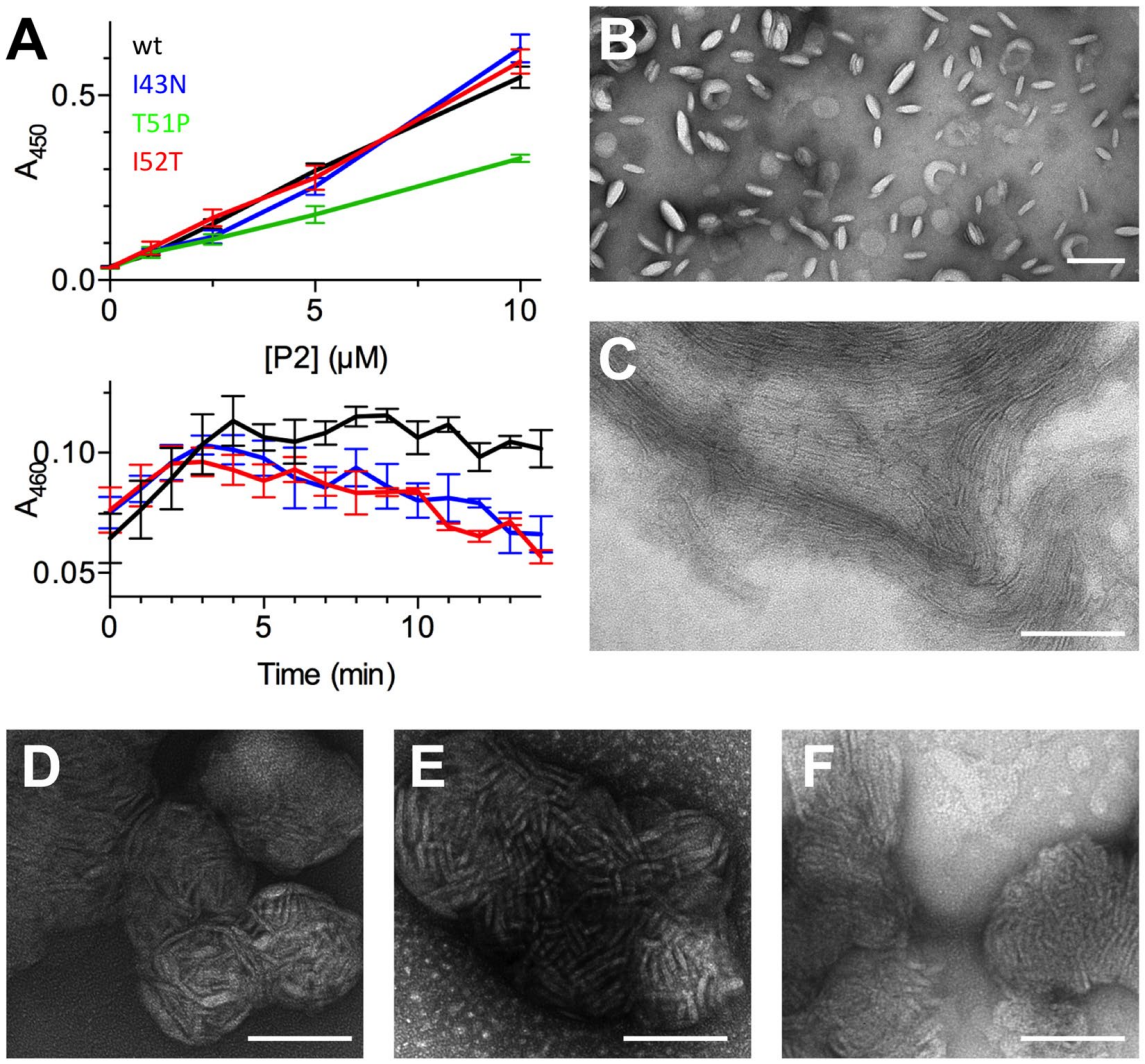
Membrane stacking by P2. (**A**) Top: end-point turbidity assay with all P2 variants. Bottom: time dependence of turbidity for P2wt and the Ile mutants. (**B–F**) Negatively stained TEM images of DMPC:DMPG vesicles (B) with P2wt (C) and mutants (D-I43N, E-T51P, F-I52T). Scale bar: 100 nm.

In compact myelin, P2 is located in the cytoplasmic leaflet, interacting with two apposing membranes. An important feature of P2 is to bind lipid membranes and to stack them into multilayered systems. To test whether the mutant variants are able to attach lipid membranes together, we used a lipid vesicle aggregation assay and measured the increase in the turbidity of a lipid vesicle solution caused by P2. The results demonstrate that both Ile mutants can bind and stack lipid membranes as efficiently as P2wt (Fig. 5A). However, T51P showed a reduced capability to stack lipid membranes; the turbidity of the vesicle-T51P mixture was approximately half of that of the other samples (Fig. 5A). With extended incubation, the Ile mutants slowly lost the turbidity, as opposed to P2wt, hinting at different stability of the proteolipid complex (Fig. 5B). We also visualised membrane stacks induced by P2 using transmission electron microscopy (TEM) and negative staining. In contrast to a control sample with only single unilamellar DMPC:DMPG vesicles (Fig. 5B), all P2 variants induced the formation of large clusters of multilamellar membrane stacks (Fig. 5C–F).

## Discussion

Recently, three P2 point mutations were shown to result in the most common heritable human peripheral neuropathy, CMT. All P2 patient mutations trigger an autosomal dominant form of demyelinating CMT1^4–6^. P2 mutations are located near each other in the structure, in close proximity to the fatty acid binding pocket and the gap between the strands β4 and β5. Comparing the sequences of all 12 human FABPs, it can be seen that the mutated residues are conserved in most human FABPs, especially those with a collision-type ligand transfer mechanism^10^. Based on our work, all CMT1-associated P2 mutant forms maintain their overall fold in the crystal state, but their stability is significantly reduced, and fatty acid and lipid membrane binding altered, compared to P2wt.

Most forms of CMT1 are caused by duplication or mutation of the *pmp22* gene, thought to lead to misfolding and aggregation of PMP22 in the ER of myelinating Schwann cells^25^. The three P2 mutations (I43N, T51P, and I52T) studied here increase the tendency of P2 to form insoluble aggregates, when expressed in *E. coli*. Even though the prokaryotic expression system differs from the natural environment inside human Schwann cells, it can provide relevant information when comparing disease-associated forms of human proteins.

The secondary structure content of T51P in solution was lower than for the other variants. All forms of P2 showed conformational changes upon lipid vesicle binding. These changes, also seen before with P2wt^10^, may originate from the α-helical lid, which interacts with the head groups of lipids and may be partially buried inside the lipid membrane. The changes could also result from a membrane-induced opening of the β barrel, leading to possible ligand exchange with the membrane. The results indicate that all P2 variants interact with lipid membranes, but as seen with SPR, the mutants are different in their respective lipid binding dynamics.

Importantly, the thermal stability of all three CMT-linked P2 variants is dramatically reduced compared to P2wt. This finding supports the results from the solubility studies; reduced protein stability leads to partial or complete unfolding and the formation of protein aggregates. Overall, the T51P mutation has the most striking effect on the biophysical properties of P2. These results raise a question whether these mutants are able to fold properly in Schwann cells, or if the mutations lead to misfolding and protein accumulation.

The crystal structures of the disease-associated mutants are highly similar to P2wt, also regarding the fatty acid binding pocket^9,10^, and differences are limited to 1–2 hydrogen bonds near the mutation site. Nevertheless, the fatty acid binding properties of all mutants differ from those of P2wt. Based on fluorescent probe assays, all three mutants bind fatty acids more effectively than P2wt, and T51P causes the highest change in DAUDA fluorescence. As DAUDA has a fairly large and rigid dansyl group at one end of the molecule, these results may suggest that T51P favours larger ligands than P2wt; the result can also reflect the increased open/close dynamics of the T51P variant. Different kinds of kinetics may come to play when the fatty acid ligand is replaced in this experiment, including affinities towards various ligands and different conformational dynamics.

RMSF analysis of MD simulations demonstrates altered dynamic properties for all disease mutants. Fatty acid-bound T51P is clearly more dynamic than P2wt and the other two mutants, and liganded T51P has more water molecules inside the β barrel than other palmitate-bound P2 variants. This is presumably the consequence of an increased flexibility of the α-helical lid and the β barrel structure of T51P, which at least to some extent arises from the lack of proper hydrogen bonds in the β sheet β1-4 (Fig. 1). The opening of the β barrel observed in the MD simulations and in solution SAXS is expected to be thermodynamically realistic, since no main-chain hydrogen bonds of the barrel need to be broken for the opening to occur. It is likely that we are, indeed, observing a functional mechanism for FABP opening, in a process that is complementary with ligand exchange. Such a mechanism was suggested earlier^26^, but to our knowledge, it has not been observed for FABPs. It is obvious that several factors may affect such conformational changes, including the bound ligand, contact to a membrane surface, and the presence of mutations. The opening of the β barrel could also be linked to misfolding and/or increased β aggregation, as observed for the T51P mutant.

A similar functional opening of a β barrel on the side has been observed for the bacterial outer membrane integral membrane protein BamA^27,28^. The barrel opens into the membrane, and the opening is believed to facilitate ligand exchange, similarly to the mechanism proposed here for myelin P2 and the FABP family. In the case of BamA, hydrogen bonds exist between the β strands in the closed state^28^, while in P2, no direct main-chain hydrogen bonds are observed even when the gap is closed. Lateral opening of the structure could present a general means of ligand exchange in β-barrel proteins.

The altered biochemical and dynamic properties of the α-helical lid, the β barrel, and the internal binding pocket of the P2 mutants may also affect the binding of other possible ligands, including cholesterol, which is bulkier than fatty acids. Cholesterol is highly abundant in myelin, and it has a rate-limiting role in myelin compaction and protein trafficking in Schwann cells^29^. Schwann cells are sensitive towards changes in the stoichiometry of their membrane components; for proper myelin assembly and especially myelin maintenance, precise amounts of different lipids are required^30^. Thus, altered fatty acid binding profiles and kinetics of membrane binding by the P2 mutants may lead to critical defects in Schwann cells and myelin stability.

P2 is localised in compact myelin, and it spontaneously stacks lipid membranes^31^. SPR showed that the mutant proteins bound to membrane surfaces, but had kinetics distinct from the wild-type protein, possibly indicating membrane-induced aggregation. Also all disease-associated forms of P2 induce the stacking of lipid membranes, as seen using TEM. A lower efficiency of the mutants to induce stable lipid membrane stacking could have severe effects on myelin compaction and stability.

Genetic knockout studies revealed changes in the lipid profile of PNS myelin in P2-deficient mice^11^, which emphasises the contribution and importance of P2 to lipid homeostasis of PNS myelin. In addition, all three CMT1-linked patient mutations disrupt the multilayered structure of myelin^4–6^. The altered stability, dynamics, and fatty acid/lipid membrane binding of the disease-associated P2 protein variants may be involved in CMT1 etiology and give rise to myelin defects, including abnormal myelin compaction and irregular myelin sheaths, in P2-linked CMT1 patients.

To conclude, we have carried out a detailed characterisation of CMT1-linked P2 mutant proteins and observed differences in their respective structures, functional properties, and dynamics. While the crystal structures of all variants are very similar, the mutant variants show clear structural changes in solution and simulations, as well as functional and stability deficits. The pathogenic point mutations drastically lower the stability of the mutant protein variants, which also have altered ligand- and membrane-binding properties. The mutations may also affect the functional dynamics of the FABP β barrel, whereby the observed opening of the gap between strands β4 and β5 may represent a general ligand entry mechanism in the protein family.

## Methods

### Mutagenesis, protein expression, and purification

Human P2 with an N-terminal His_6_ tag followed by a Tobacco Etch virus (TEV) protease cleavage site in the pTH27 vector^9^ was used as a template in mutagenesis to produce expression constructs with the c.T128A (p.I43N), c.C151A (p.T51P), and c.T155C (p.I52T) mutations. Mutagenesis was carried out using the QuikChange Site-Directed Mutagenesis protocol, and all mutations were confirmed by DNA sequencing. Proteins were expressed in *E. coli* BL21 RIPL (DE3) cells in ZYM-5052 autoinduction medium^32^ with 100 µg/ml carbenicillin and 34 µg/ml chloramphenicol at +18 °C for 66 h. Cells were harvested and suspended in lysis buffer (0.3 M NaCl, 10 mM imidazole, 50 mM HEPES pH 7.5). Cells were lysed by sonication, and insoluble material was pelleted by centrifugation (30000 g, 40 min, at +4 °C). The soluble fraction was then mixed with the HisPur Ni-NTA Resin (Thermo Fisher Scientific) at +4 °C for 1 h. The resin was washed twice with washing buffer (0.3 M NaCl, 40 mM imidazole, 50 mM HEPES pH 7.5) using centrifugation (500 g, 5 min, at +4 °C) and then transferred into a gravity-flow column and further washed with 50 ml of washing buffer. P2 was eluted with elution buffer (500 mM imidazole, 0.3 M NaCl, 50 mM HEPES pH 7.5). To cleave the His_6_ tag, 33 nmol of recombinant TEV protease^33^ were added. Imidazole was removed by dialysis through 6000–8000 MWCO dialysis tubing (SpectraPor) against 0.3 M NaCl, 1 mM DTT, 20 mM HEPES pH 7.5 at +4 °C for 16 h. Both TEV and the cleaved His_6_ tag were removed with reverse chromatography using HisPur Ni-NTA. Finally, P2 was purified with SEC using the dialysis buffer and a Superdex 75 pg 16/600 column (GE Healthcare) and concentrated with an Amicon Ultra 15, MWCO 10 kDa concentrator (Millipore). In T51P purification, all buffers, excluding the SEC buffer, also contained 10% glycerol to maintain the protein soluble during purification.

### Protein crystallisation, data collection, and structure determination

P2 mutants were crystallised at +4 or +20 °C using the sitting drop vapour diffusion method, while the concentrations of the I43N, T51P, and I52T mutants were 8.5, 6.2, and 5.5 mg/ml, respectively. 0.3 M NaCl, 10% glycerol, 20 mM HEPES pH 7.5 was used as the protein buffer and 100 µl of 2.1 M DL-malic acid pH 7.25–7.5 as the reservoir solution, with 600-nl drops containing an equal volume of reservoir and protein. Diffraction data were collected at 100 K on beamline I03 and I04 at the Diamond Light Source, Didcot, UK, and processed using XDS^34^. The structures were solved by molecular replacement using the human P2 structure (PDB entry 4BVM^10^) as a search model in Phaser^35^. The structures were refined and built using phenix.refine^36^ and Coot^37^. The refined coordinates and structure factors were deposited at the PDB with the entry codes 5N4M (I43N), 5N4P (I52T), and 5N4Q (T51P).

### Small-angle X-ray scattering

Synchrotron SAXS data were collected on beamline P12 at the PETRAIII storage ring, DESY, Hamburg, Germany. 0.6–4.1 mg/ml P2wt, I43N, and I52T were studied in a buffer containing 0.3 M NaCl, 20 mM HEPES pH 7.5, and T51P in a buffer containing 0.3 M NaCl, 5% glycerol, 20 mM HEPES pH 7.5. Data were processed and further analysed using the ATSAS program package^38^.

### Protein aggregation analysis and dynamic light scattering

Samples from soluble and insoluble fractions were taken and run on SDS-PAGE. Intensities of the P2 protein bands were determined using ImageJ^39^. Soluble fractions were then calculated (%). DLS measurements were conducted as triplicates at +25 °C with protein concentrations of 0.6–0.9 mg/ml in a buffer containing 0.3 M NaCl, 20 mM HEPES pH 7.5, using the DynaPro Plate Reader II (Wyatt). After SEC, the proteins were stored on ice for 24 h before DLS measurements.

### Circular dichroism spectroscopy

SRCD was carried out on the DISCO (SOLEIL synchrotron, Paris) and UV-CD12 (ANKA synchrotron, Karlsruhe) beamlines. Proteins were first dialysed into 10 mM sodium phosphate pH 7.5 or dH_2_O. SRCD spectra were measured at +30 °C using 0.2 mm pathlength quartz cuvettes with protein concentrations of 17–21 µM (~0.3 mg/ml). Spectra for the P2 variants were also recorded in the presence of unilamellar vesicles containing a 1:1 molar ratio of DMPC and DMPG, or DOPC and DOPG. The molar protein:lipid ratio was 1:100. Thermal stability measurements were carried out using a Chirascan Plus CD spectropolarimeter (Applied Photophysics). Proteins were first diluted into 10 mM sodium phosphate buffer (pH 7.5) to final concentrations of 3.4 µM (50 µg/ml). CD spectra were then recorded at 195–260 nm with a ramping rate of 1 °C/min between +22–90 °C. The melting temperatures (T_m_) were calculated using Global 3 (Applied Photophysics).

### Atomistic molecular dynamics simulations

The protein with bound palmitate was obtained from the PDB entry 4BVM^10^ and converted to the CHARMM36 force field^40^. The topology for P2wt was directly obtained from the conversion. The point mutations (I43N, T51P, and I52T) were constructed from P2wt. Water molecules were modeled using the TIP3P model^41^.

Eight different systems were studied: P2wt and its point-mutated forms I43N, T51P, and I52T, all with and without a bound palmitate molecule inside the binding pocket. The proteins were simulated in solvated cubic simulation boxes with sizes of approximately 8 × 8 × 8 nm^3^ and with 16000 water molecules, in accordance to our previous study^42^. Counter-ions (11 Cl^−^ in palmitate-free proteins and 10 Cl^−^ in proteins with palmitate) were included to neutralise the total charge of the system.

The MD simulations were carried out under NpT conditions. Temperatures were coupled using the velocity-rescale (v-rescale) method, with separate temperature coupling for protein and solvent. Reference temperatures of 300 K were used with coupling time constants of 2.0 ps. Pressure coupling was done isotropically with the isothermal Parrinello-Rahman barostat^43^ at a reference pressure of 1 bar with a coupling time constant of 2.0 ps and isothermal compressibility of 4.5 × 10^−5^ bar^−1^. All bonds were constrained with the LINCS algorithm^44^. Periodic boundary conditions were used. A cut-off radius of 1.0 nm was introduced for the neighbor list, the Lennard-Jones interactions, and the non-bonded interactions. The particle-mesh Ewald (PME) method^45^ with cubic interpolation was used for calculating long-range electrostatics, using a spacing of 0.16 nm for the Fourier grid.

All simulations were conducted using GROMACS 4.6.7^46^. A time step of 2 fs was used in integrating the equations of motion. All systems were first energy-minimised with the steepest descent algorithm and then simulated for a total of 2.5 µs each. An equilibration period of 500 ns was removed from the beginning of each trajectory, using the final 2 µs for analysis. The trajectory coordinates were saved every 50 ps.

The RMSF was calculated for each residue using the GROMACS tool g_rmsf. The RMSF of a residue shows its stability; the larger the value, the more mobile it is. Therefore, by calculating the RMSF, we can determine, which parts of the protein are most affected by the mutation and/or the presence of the ligand. Water analysis was done with the GROMACS tool trjorder by calculating the number of water molecules within 0.9 nm of the center of mass of the protein backbone (the radius of the P2 barrel is about 1.0 nm) at every time step over the course of the simulation.

### Fatty acid binding assay

Fatty acid binding to P2 was studied with a fluorescent fatty acid analog, DAUDA. 10 µM DAUDA and 10 µM P2 were incubated for 1 h in 150 mM NaCl, 10 mM HEPES pH 7.5 at +23 °C. Fluorescence emission spectra were recorded with excitation at 345 nm and emission between 400–700 nm, using a Tecan Infinite M1000Pro plate reader.

### Liquid chromatography-mass spectrometry for fatty acids

20-µl aliquots of protein solutions adjusted to the same concentration (30 µM; 0.45 mg/ml) were acidified with 1 µl formic acid and then precipitated with 20 µl of acetone. Samples came from 2–3 separate production batches for each variant, and each sample was analysed 1–2 times. After centrifugation, fatty acids were extracted into 20 µl of chloroform, the phases were separated by short centrifugation, and 5 µl of the chloroform phase was subjected to LC-MS using an Acquity UPLC system coupled to a SynaptG1 Q-TOF type mass spectrometer. The chromatography column was a BEH C18, 2.1 × 100, eluted with a gradient of acetonitrile (A: acetonitrile, B: 10 mM ammonium acetate 10% A to 100% A in 8 min, flow 0.3 ml/min). The mass spectrometer was operated in negative mode, collecting 0.2-s scans in centroid mode from m/z 50 to 1000. LC-MS was performed at the Proteomics and protein analysis core facility of Biocenter Oulu.

### Surface plasmon resonance

SPR assays were performed using BiacoreT200 (GE Healtcare) for P2wt as well as the I43N and I52T mutants. Due to its low stability and tendency to aggregate, T51P was not used in SPR. 1 mM DOPC:DOPG (1:1) and DMPC:DMPG (1:1) vesicles in a buffer containing 150 mM NaCl and 10 mM HEPES pH 7.5 were immobilised on an L1 sensor chip (GE Healthcare) according to the manufacturer’s instructions. The surface was then saturated with an injection of 1 µM bovine serum albumin (BSA). Lipid immobilisation and BSA injection were done for each SPR cycle. Duplicate injections of P2wt and the mutants at 0.1–10 µM, using 150 mM NaCl, 10 mM HEPES pH 7.5 as the running buffer, were carried out at +30 °C. Results were analysed with BiaEvaluation (GE Heathcare) using a kinetic two-state binding model as well as steady-state affinity. Due to the shape of the binding curves, fitting was also done using a sigmoidal 4-parameter model

1 $$f={y}_{0}+\frac{a}{(1+\exp (-(x-{x}_{0})/b))}$$

in SigmaPlot.

### Vesicle binding assays

For a single-point assay, 0–10 µM P2 was mixed with 0.5 mM unilamellar vesicles containing a 1:1 molar ratio of DMPC:DMPG in 150 mM NaCl, 10 mM HEPES pH 7.5, and the samples were incubated for 35 min at +30 °C. The turbidity of the vesicle-protein solutions was measured with a Tecan Infinite M1000 Pro plate reader using absorbance at 450 nm at +30 °C. The assay was repeated 3–5 times with all concentrations and P2 forms.

In order to study the time course of vesicle aggregation, 0.5 mM DMPC:DMPG vesicles were mixed with 5 µM P2 in buffer containing 150 mM NaCl and 10 mM HEPES (pH 7.5). A VersaMax microplate reader (Molecular Devices) was used to measure the turbidity at 1-min intervals, shaking before each measurement. The temperature was set at +30 °C and the wavelength at 460 nm. The difference of 10 nm in the wavelength in the above two experiments was not considered to affect the outcome, as the measurement follows turbidity of the sample.

### Transmission electron microscopy

34 µM (0.5 mg/ml) P2 was mixed with 740 µM (0.5 mg/ml) DMPC:DMPG (1:1) unilamellar vesicles, and the samples were incubated at +22 °C for 1 h. 4-µl samples were then pipetted onto glow-discharged carbon-coated copper grids, and after incubating for 1 min, excess solution was removed with filter paper. After 4 washes in droplets of dH_2_O, samples were negatively stained with two drops of 2% uranyl acetate for 12 s in each drop and air-dried. TEM images were recorded using a Tecnai G2 Spirit 120 kV instrument equipped with a Quamesa CCD camera (Olympus Soft Imaging Solutions) at the EM core facility of Biocenter Oulu.

### Data availability

The crystal structure coordinates and structure factors are available at the PDB with the entry codes 5N4M (I43N), 5N4P (I52T), and 5N4Q (T51P). Other datasets analysed during the current study are available from the corresponding author upon reasonable request.
