# Publisher Correction: Molecular mechanisms of Charcot-Marie-Tooth neuropathy linked to mutations in human myelin protein P2

**DOI:** 10.1038/s41598-017-18751-7

**Published:** 2018-01-08

**Authors:** Salla Ruskamo, Tuomo Nieminen, Cecilie K. Kristiansen, Guro H. Vatne, Anne Baumann, Erik I. Hallin, Arne Raasakka, Päivi Joensuu, Ulrich Bergmann, Ilpo Vattulainen, Petri Kursula

**Affiliations:** 1 Faculty of Biochemistry and Molecular Medicine, University of Oulu, 90220 Oulu, Finland; 2 Department of Physics, Tampere University of Technology, 33720 Tampere, Finland; 3 Department of Biomedicine, University of Bergen, 5020 Bergen, Norway; 4 Division of Psychiatry, Haukeland University Hospital, 5021 Bergen, Norway; 5 Department of Sustainable Chemistry, Technical Faculty, University of Oulu, 90570 Oulu, Finland; 6 Biocenter Oulu, University of Oulu, 90220 Oulu, Finland; 7 Department of Physics, University of Helsinki, 00560 Helsinki, Finland

Correction to: *Scientific Reports* 10.1038/s41598-017-06781-0, published online 26 July 2017

The PDF version of this Article was inadvertently replaced after publication. This has now been corrected; the HTML version of the paper was correct from the time of publication.

